# Transient Bilateral Atrial Thrombi in a Patient With Multiple Sclerosis and Massive Pulmonary Embolism: A Case Report and Review of the Literature

**DOI:** 10.7759/cureus.87998

**Published:** 2025-07-15

**Authors:** Mae Hands, Abhimanyu J Baruah, Kelvin Lam, Syed Mustafa, Nirishka Seewoosungkur, Adnan Ahmed, Jhiamluka Solano

**Affiliations:** 1 Emergency Medicine, Scunthorpe General Hospital, Scunthorpe, GBR; 2 Acute Medicine, Northern Lincolnshire and Goole NHS Foundation Trust, Scunthorpe, GBR; 3 Internal Medicine, Northern Lincolnshire and Goole NHS Foundation Trust, Scunthorpe, GBR; 4 Cardiology, Northern Lincolnshire and Goole NHS Foundation Trust, Scunthorpe, GBR; 5 Cardiology, Hull University Hospital (Castle Hill Hospital), Hull, GBR; 6 Resident Doctor Committee, Royal College of Physicians, London, GBR; 7 Education Committee, Academy of Medical Educators, Cardiff, GBR; 8 Cardiology, Scunthorpe General Hospital, Scunthorpe, GBR

**Keywords:** atrial septal defect, embolic stroke, intracardiac thrombus, multiple sclerosis, pulmonary embolism

## Abstract

Pulmonary embolism (PE) can present with complex and atypical features that challenge diagnosis and management, particularly when coexisting with intracardiac thrombi and neurological symptoms. We report a case of a 67-year-old woman with multiple sclerosis (MS) who presented with bilateral PE, bi-atrial thrombi, and a subsequent embolic stroke. Despite initial imaging suggesting a possible atrial septal defect (ASD) as the route for paradoxical embolism, both transthoracic echocardiography and cardiac magnetic resonance imaging failed to confirm any intracardiac shunt. The case underscores the diagnostic difficulties in differentiating between stroke and MS relapse, as well as limitations of imaging modalities in transient thrombotic or shunt-related phenomena. The patient was managed with therapeutic anticoagulation, which resulted in thrombus resolution and clinical improvement. This case highlights the need for high clinical suspicion and multidisciplinary evaluation in patients with overlapping cardiovascular and neurological presentations, especially in the setting of chronic inflammatory diseases such as MS.

## Introduction

Pulmonary embolism (PE) is a potentially life-threatening condition often resulting from deep venous thrombosis and characterised by variable clinical presentation. Rarely, thrombi may be visualised in transit within the cardiac chambers, particularly the right atrium, which poses a high risk for further embolisation and is associated with increased mortality [1]. The differential diagnosis for intracardiac masses includes thrombi, myxomas, and other space-occupying lesions. The coexistence of intracardiac thrombi, systemic embolic events, and suspected intracardiac shunts such as an atrial septal defect (ASD) or patent foramen ovale (PFO) adds significant diagnostic complexity [2]. In patients with underlying neurological conditions such as multiple sclerosis (MS), overlapping symptomatology may further complicate the clinical picture. We present the case of a 67-year-old woman with MS who developed bilateral PE, transient intracardiac thrombi, and neurological deficits, with imaging ultimately excluding the presence of ASD or PFO. This case is of particular interest due to the rare combination of paradoxical embolic phenomena in the absence of an identifiable intracardiac shunt, raising important diagnostic and pathophysiological considerations.

## Case presentation

A 67-year-old woman with a background of MS and a known smoker presented to the emergency department with a one-week history of worsening constipation, which progressed to absolute constipation, accompanied by a firm, distended, and non-tender abdomen and a new oxygen requirement.

Upon examination, she was tachycardic (HR 140 bpm), tachypneic (RR 24), with oxygen saturations of 97% on 15L of oxygen via a non-rebreather mask, normothermic (36.5°C), and normotensive (BP 121/80 mmHg). Initial laboratory investigations showed an elevated C-reactive protein (CRP) of 237 mg/L, white cell count (WCC) of 14.4x10⁹/L, serum bilirubin of 27 µmol/L, alanine aminotransferase (ALT) of 152 U/L, and hyponatremia with a sodium level of 126 mmol/L (see Table 1).

**Table 1:** Initial blood test.

Test	Result	Reference range	Interpretation
C-reactive protein (CRP)	237 mg/L	<5 mg/L	Markedly elevated – inflammation/infection
White cell count (WCC)	14.4 ×10⁹/L	4.0–11.0 ×10⁹/L	Elevated – leukocytosis
Serum bilirubin	27 µmol/L	<21 µmol/L	Mildly elevated – possible liver dysfunction or hemolysis
Alanine aminotransferase (ALT)	152 U/L	<40 U/L	Elevated – hepatocellular injury
Sodium (Na⁺)	126 mmol/L	135–145 mmol/L	Hyponatremia – moderate

**Figure 1:**
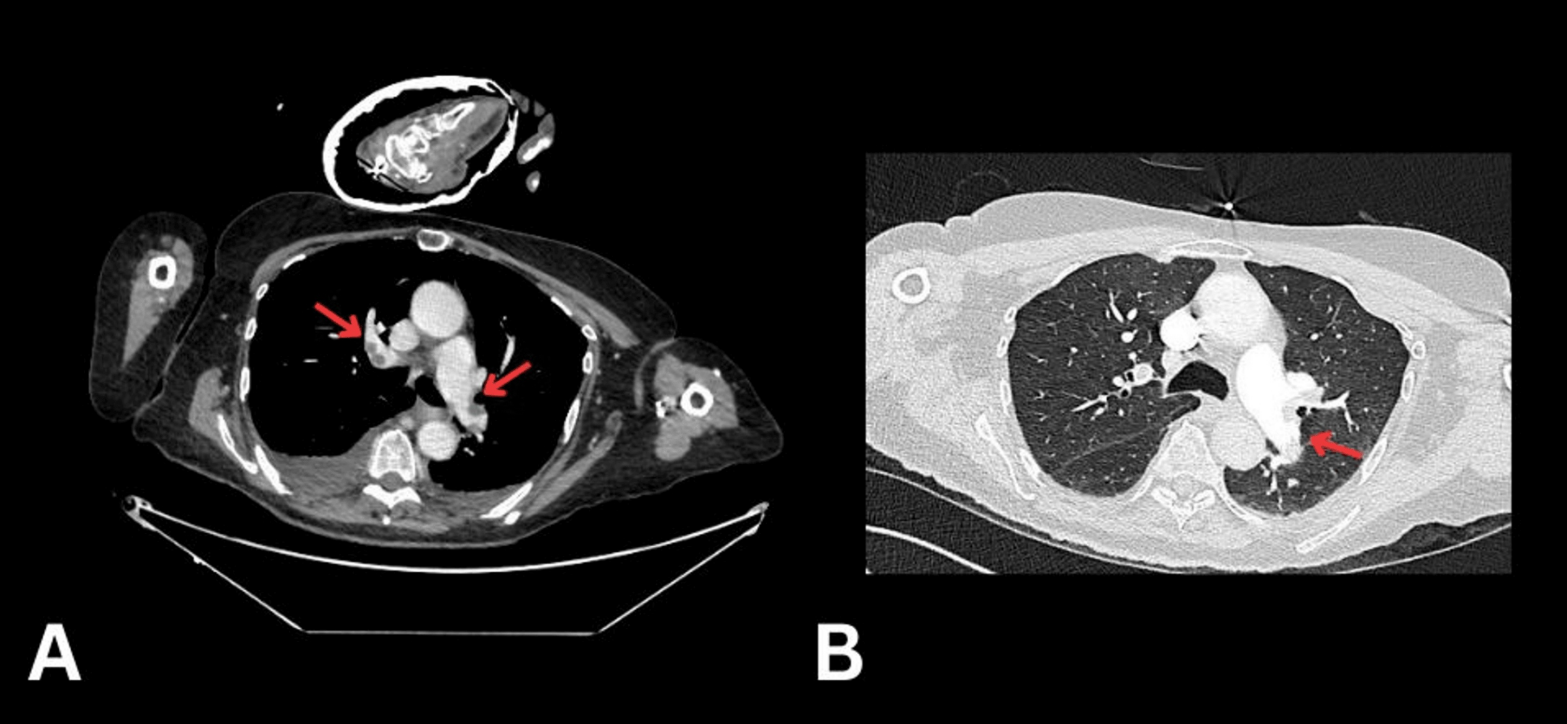
Computed tomography of the chest, abdomen, and pelvis (CT PA). The arrows indicate the presence of thrombus in the pulmonary circulation.

A computed tomography of the thorax, abdomen, and pelvis (CT TAP), performed to investigate potential bowel obstruction and the cause of her oxygen requirement, revealed a large burden of pulmonary emboli involving thrombus in the right main, truncus anterior, right upper lobe, middle lobe, interlobar, and right lower lobe pulmonary arteries, as well as thrombus in the left main, left upper lobe, lingular, interlobar, and left lower lobe pulmonary arteries (see Figure 1A, 1B). There was no radiological evidence of right heart strain. Additional findings included bi-basal patchy opacification consistent with infection, dilated ascending thoracic aorta and pulmonary arteries, marked bilateral hydronephrosis with ureteric dilation to the bladder ostia, mild bladder wall thickening causing upstream obstruction, and marked dilatation of the urinary bladder. A marked colonic faecal burden was also noted.

She was commenced on treatment-dose enoxaparin and referred to the cardiology team. An inpatient bedside transthoracic echocardiogram (TTE) revealed a large, highly mobile, space-occupying lesion involving both the left and right atria, appearing attached to the interatrial septum. The right heart was dilated, with severe tricuspid regurgitation and a Doppler estimated pulmonary artery systolic pressure (PASP) estimated at >74 mmHg (reference range: 18-25 mmHg). There was concern for a possible ASD (see Video 1).

**Video 1:** Transthoracic echocardiogram (TTE). The arrows show the presence of thrombus in both atria. A. Parasternal long axis view (PLAX). B. Apical four-chamber with RV dedicated view. C. Apical four-chamber view. D. Apical four-chamber with volume rendering colour map.

**Figure 2:**
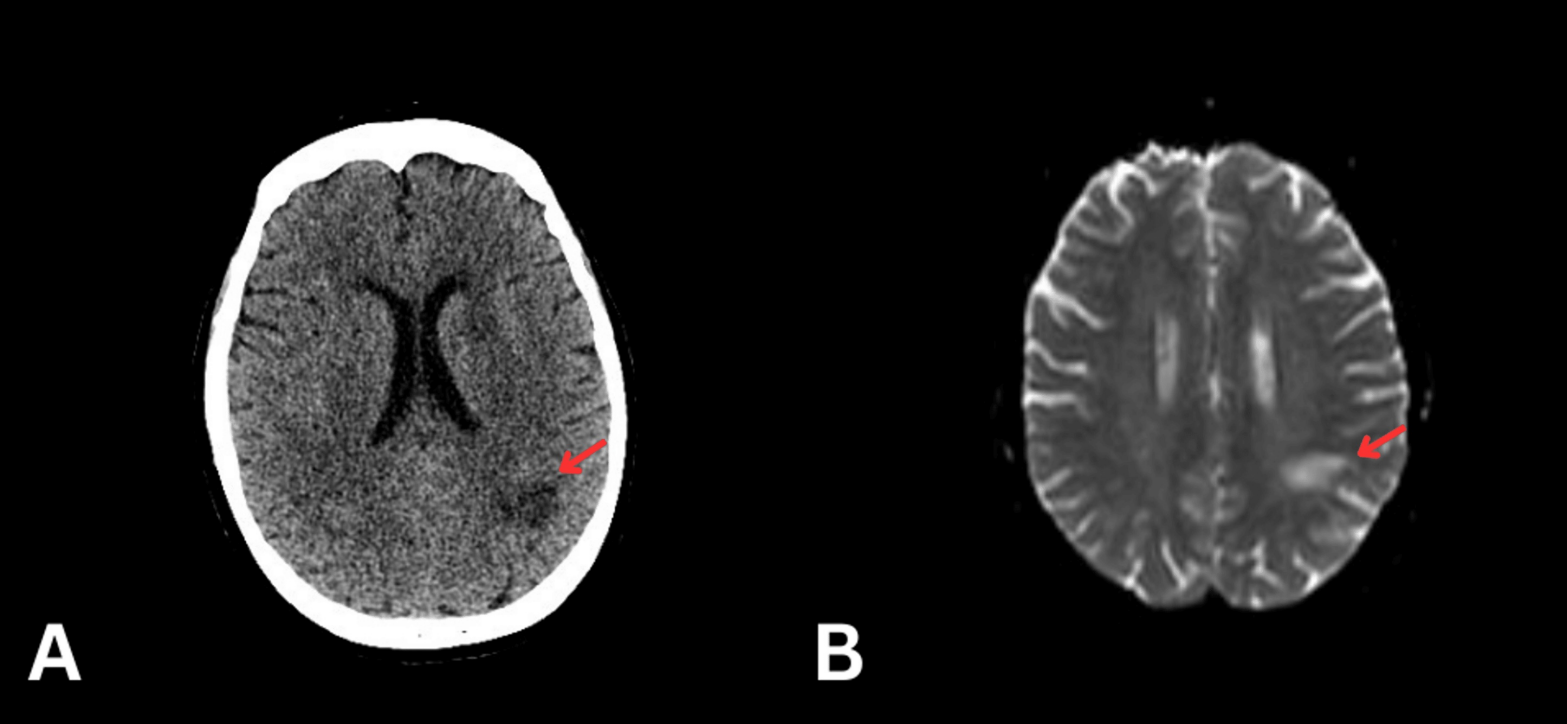
CT head and MRI head. The arrows show the left parietal lobe changes. A. CT head demonstrated left parietal lobe changes, which were considered either demyelination or subacute infarction. B. MRI of the brain confirmed infarction using diffusion-weighted imaging (DWI) and apparent diffusion coefficient (ADC) mapping.

Two weeks after admission, she developed new left-sided weakness, raising concerns for a cerebrovascular event. A CT head demonstrated left parietal lobe changes, which were considered either demyelination or subacute infarction (see Figure 2A). Subsequent MRI of the brain confirmed infarction, but noted that the findings did not correlate with the side of the patient’s clinical weakness (see Figure 2B). MRI sequences included diffusion-weighted imaging (DWI), apparent diffusion coefficient (ADC) mapping, T2-weighted imaging, FLAIR, SWI, and T1-weighted imaging. In addition, it was described that some of the new white matter lesions demonstrate DWI signal changes but no significant ADC changes. These were concluded to be chronic microembolic ischemic cerebral infarcts.

A repeat TTE a few days later was performed to reassess intracardiac thrombus burden and evaluate for the presence of ASD. No thrombus was visualised in the left or right atrium, and no definitive ASD was identified, although its presence could not be excluded. The right heart remained dilated but demonstrated normal systolic function. PASP had reduced to 27-32 mmHg. The patient was subsequently transferred to a tertiary centre for cardiac magnetic resonance imaging (MRI) to investigate the aetiology of the intracardiac thrombi and to assess for an ASD. Cardiac MRI showed no evidence of an ASD or patent foramen ovale (PFO) and no residual intracardiac thrombus or space-occupying lesion (see Video 2). 

**Video 2:** MRI heart.

The patient was started on rivaroxaban 15 mg BD and then 20 mg OD. While lifelong anticoagulation was deemed necessary given the recurrence risk, the bleeding risk was weighed carefully, particularly considering the patient’s MS-related mobility limitations and potential for trauma. Shared decision-making involved evaluating the HAS-BLED (Hypertension, Abnormal Renal/Liver Function, Stroke, Bleeding History or Predisposition, Labile INR, Elderly, Drugs/Alcohol Concomitantly) score and patient preference.

## Discussion

This case illustrates a rare and complex presentation of bilateral PE with bilateral intracardiac thrombi and an embolic stroke with an underlying diagnosis of MS in the absence of any evidence of an intracardiac shunt, such as an ASD or patent foramen ovale. Each of these findings individually can carry significant morbidity and mortality, and their coexistence in a single patient poses substantial diagnostic and management challenges.

The simultaneous presence of thrombi in both the right and left atria is highly atypical. Right atrial thrombi typically originate from venous thrombosis and may embolise to the lungs, while left atrial thrombi are usually associated with atrial fibrillation or a structural abnormality [3]. Their coexistence suggests either a paradoxical embolism through a right-to-left shunt, which is rare in a patient in sinus rhythm; however, the diagnostic challenge was that no ASD or PFO was identified. In addition, MS adds a layer of complexity to this case as it has been associated with an increased risk for venous thromboembolism (VTE) [4].

The range of differentials for intracardiac thrombi is limited (see Table 2), but it adds a layer of complexity to the clinical presentation of our patient [5]. However, high-risk features found in patients with bi-atrial thrombi in the absence of a PFO include severe left ventricular dysfunction (EF < 30%), aortic or mitral valve disease, or a history of atrial fibrillation [3].

**Table 2:** Range of differentials for intracardiac thrombi Adapted from Alkindi, Hamada, and Hajar, 2013 [5].

Differential	Distinguishing feature	Key imaging modality
Myxoma	Septal attachment, vascularity	MRI / contrast echo
Chiari network	Filamentous, mobile	TEE
Atrial septal aneurysm	Septal bulging, not free-floating	TEE
Catheter-related	Known lead/catheter, echogenic line	X-ray, CT
Crista terminalis	Fixed, anatomic	TEE
Lipomatous hypertrophy	Dumbbell shape, septal	MRI / CT
Artifact	View-dependent	Multiple echo views
Vegetation	Infective signs, valve involvement	Echo + clinical context

The patient’s initial presentation with bilateral pulmonary emboli, a large mobile intracardiac mass involving both atria and subsequent cerebral infarction raised strong clinical suspicion for an underlying right-to-left shunt facilitating embolic stroke. However, despite repeated transthoracic echocardiography (TTE) and cardiac magnetic resonance imaging (MRI), no definitive shunt or residual thrombus was identified. This highlights the limitations of standard imaging modalities, particularly in detecting small or transient shunts, as well as the potential impact of patient factors and the transient nature of embolic phenomena [6,7]. Moreover, the discordance between neurological symptoms and imaging findings underscores the diagnostic challenge posed by coexisting pathologies, such as MS, which complicate the clinical picture [8].

Management of intracardiac thrombi and embolic stroke without confirmed shunt primarily relies on anticoagulation, which was appropriately initiated in this patient and likely contributed to thrombus resolution and improvement in pulmonary pressures. Current evidence and guidelines recommend anticoagulation in an embolic stroke of an undetermined source (ESUS) only when a cardioembolic cause, such as atrial fibrillation or shunt, is identified [9,10]. However, mounting evidence has implicated MS as a potential risk factor for VTE, with multiple epidemiological studies and meta-analyses highlighting an elevated incidence of deep vein thrombosis (DVT) and PE in this population [11]. A study by Christensen et al. [12] revealed that the increased risk was most pronounced during the first year following the initial diagnosis but persisted at a moderately lower level for up to 30 years. This heightened risk applied to both PEs and DVTs and was quasi-equivalent for provoked and unprovoked VTEs. It should be noted that although there was robust evidence to support an increased risk of VTE among MS patients, the study demonstrated that the absolute risk remains relatively low. A meta-analysis performed by Ghoshouni et al. [11] suggested that MS patients’ risk of VTE is more than doubled when compared to their reference population, with an incidence of 0.9% of MS patients developing PEs. This contrasts an early study performed in 1988 by Kaufman et al. [13], which reported no increased risk of VTE relative to patients hospitalised for other conditions. The authors posited that spastic paralysis, characteristic of MS, might preserve venous return more effectively than the flaccid paralysis seen in spinal cord injury, thus mitigating venous stasis and its thrombotic sequelae. More recent studies, such as the research by Peeters et al. [4], have refuted this hypothesis, finding that muscle spasticity is associated with a 2.6-fold increased risk of VTE among MS patients.

The pathophysiology behind VTE formation involves a triad of venous stasis, hypercoagulability, and endothelial damage. In MS, patients will often suffer from reduced mobility as a result of neurological deterioration, with immobilisation being a clear and well-established risk factor for VTEs. This hypothesis is corroborated by the study by Christensen et al. [12], which demonstrated a correlation between an increased risk of VTE and the number of MS-related hospitalisations. MS is an autoimmune condition, and as such, it displays features of chronic inflammation, leading to the upregulation of procoagulant pathways and the disruption of endothelial homeostasis, which facilitates thrombus formation. Furthermore, subtype-specific analyses conducted by Roshanisefat H et al. [14] demonstrated that patients with primary progressive and secondary progressive MS exhibit a significantly greater propensity towards VTE compared to those with the relapsing-remitting form, likely a reflection of immobilisation being a more prominent feature of the more aggressively progressive forms of MS. Complimentarily, a meta-analysis by Hong et al. [15], extends findings by evidencing increased risk of ischaemic stroke in MS patients, attributed to overlapping mechanisms including endothelial damage and accelerated atherogenesis - pathophysiological processes that mirror those involved in VTE formation.

The patient’s new left-sided weakness raised concerns towards a neurological pathology; thus, an MRI diffusion-weighted brain was done. However, the results proved to be a diagnostic challenge as the differential diagnosis in this context was a possible MS relapse, given the background of MS or an acute stroke, because of the atrial thrombi. The MRI showed a patchy subacute cortical and subcortical infarct of the left parietal lobe, which corresponds to an infarct secondary to the atrial thrombi and prothrombotic state of the patient, but the lesion's anatomical site did not correspond to the patient’s left-sided weakness as this neurological deficit should be contralateral to the brain lesion. 

The MRI also revealed bilateral deep white matter high-signal changes in the periventricular area, which had increased compared to the patient’s previous MRI in 2021, potentially indicating an MS flare-up. MS lesions tend to present in the periventricular regions and are consistent with the McDonald diagnostic criteria for MS, which require one or more T2 hyperintense lesions in at least 2 of the following areas: periventricular, cortical, or juxtacortical, infratentorial brain regions, or the spinal cord [16]. However, distinguishing between MS and stroke based solely on the lesion’s anatomical location can be difficult. 

Diffusion-weighted imaging (DWI) and apparent diffusion coefficient (ADC) values are useful in distinguishing an acute stroke from an MS flare. In MS, lesions often show high DWI and high ADC values due to vasogenic oedema, where inflammation leads to fluid accumulation in the extracellular space. By contrast, acute or subacute strokes typically present with high DWI and low ADC values, reflecting cytotoxic oedema from intracellular swelling [17]. In our case, the new periventricular lesions showed DWI hyperintensity without significant ADC reduction, a pattern more consistent with subacute ischaemic changes, particularly in the context of known atrial thrombi. However, there could also be early transient ADC reduction at the very early stage of MS lesions, which may cause new symptoms [18].

One of the limitations was that the MRI was done without contrast. Gadolinium contrast will enhance lesions showing active inflammatory lesions of MS [19]. This could have given us more information to differentiate between an MS flare and an acute stroke. MS flare-ups can mimic a stroke, hence the diagnostic challenge; however, the symptoms of MS usually worsen over hours and days, whereas stroke occurs suddenly within minutes. In this case, given the radiological findings and the clinical context that the patient’s left-sided weakness occurred suddenly but during admission got progressively worse, then improved within 1 week, the main differential was a relapse of the MS.

According to the current National Institute for Health and Care Excellence (NICE) guidelines, there are five mainstay pharmacological treatments for a confirmed PE: fondaparinux, low-molecular-weight heparin (LMWH) followed by oral anticoagulants, oral anticoagulants (such as warfarin, apixaban, and rivaroxaban), or unfractionated heparin [20]. The patient, in this case, was started on rivaroxaban 15 mg twice a day for 12 days and then 20 mg once a day thereafter. This is in line with current guidance, which recommends offering either apixaban or rivaroxaban to people with confirmed proximal DVT or PE in patients without specific comorbidities [21], none of which this patient had. In terms of treatment duration, NICE guidelines suggest a 6-month course of rivaroxaban, considering that this PE would be classified as unprovoked. However, as discussed in NICE's evidence-based review on rivaroxaban, clinical specialists may consider lifelong anticoagulation in patients who have had a massive pulmonary embolus, recurrent VTE or are deemed to be at significant risk of recurrence [21]. The presence of bilateral, unprovoked PEs in this patient supported lifelong anticoagulation. However, with any anticoagulation regimen, it is crucial to consider the patient's bleeding risk. Patients with MS have an increased risk of developing strokes, including hemorrhagic strokes [15,22].

Lessons learned, clinical implications, and future considerations

Patients with MS face nearly double the risk of VTE compared to the general population, alongside an elevated risk of intracerebral haemorrhage (ICH) and acute ischaemic stroke. These risks necessitate a careful, individualised risk-benefit analysis when considering anticoagulation, particularly in the presence of contributing factors such as tobacco use. The potential protective role of disease-modifying therapies (DMDs) should also be considered. Diagnostic complexity in this population underscores the need for meticulous baseline documentation and the use of multimodal imaging to detect neurological deterioration promptly in the context of challenging or overlapping clinical presentations. Further epidemiological research is essential to validate these findings and guide more effective management strategies for patients with MS.

Patient’s perspective

‘I have MS, and that affects every part of my body. This first started when I fell and broke my wrist, leading me to the hospital. While I awaited surgery, I was transferred to a care home for a week. There, I was left in bed all the time, which led to a worsening of my mobility. Eventually, I was back in the hospital for my operation. One day, I asked a staff member to help me go to the bathroom, and I told her I needed assistance with two tasks. She said she felt confident she could help me by herself. This led to another fall, which made the staff worried about me, resulting in more time spent restricted to bed. After the operation, I was sent to another care home. Here again, I stayed in bed most of the time, leading to bed sores. I found myself back in the hospital because I did not feel right, and various scans found blood clots in my heart and lungs and a stroke I had no idea about. I was admitted to the cardiology ward. This ward was run exceptionally well. I received excellent care and benefited from the physiotherapy team's assistance. I was explained my diagnosis well, and I feel the staff did everything they could to investigate my condition. However, I felt my MS was ignored, and only the clots were thought about. While the physiotherapy on the ward helped, my mobility was still very poor, so I was sent to a rehabilitation facility. Here, the staff were able to provide me with the one-on-one time I needed. They have put a lot of effort into my recovery, and I am much improved from the initial fall; however, I am still not at my baseline. I am going back to my own home next week, which I am very excited about. 

Overall, I do believe more could have been done so that I was not in bed for so long. I feel that this played a role in my deteriorating mobility, alongside the falls and the progression of my MS. I think that, as much as the hospital staff want to support me, there simply isn't enough staff. There were not enough people to give me the 1:1 time that I needed. I also do not understand why the cardiac MRI was done for me, as I was told that even if something were found, I would not be fit enough for surgery. Given all of this, I am still excited to be able to go back to my home, and I hope my case provided some learning points to someone reading.’

## Conclusions

This case aims to illustrate the unique diagnostic interplay between cardiopulmonary thromboembolism, transient intracardiac masses, and unexplained cerebral infarction in a patient with MS, where multimodality imaging ultimately excluded an intracardiac shunt, raising important questions about stroke pathophysiology in proinflammatory neurological disease. Given the patient's unprovoked bilateral pulmonary embolism, transient intracardiac thrombi, and embolic cerebral infarct, long-term anticoagulation was deemed appropriate. While no intracardiac shunt was identified, the constellation of findings suggested a high thromboembolic risk. In the context of chronic neurological disease, systemic inflammation and functional immobility due to MS, indefinite anticoagulation was considered as a secondary prevention strategy, acknowledging the lack of formal guidelines in patients with MS and unprovoked embolic events.
